# Cerevisterol Alleviates Inflammation via Suppression of MAPK/NF-κB/AP-1 and Activation of the Nrf2/HO-1 Signaling Cascade

**DOI:** 10.3390/biom10020199

**Published:** 2020-01-29

**Authors:** Md Badrul Alam, Nargis Sultana Chowdhury, Md Hossain Sohrab, Md Sohel Rana, Choudhury Mahmood Hasan, Sang-Han Lee

**Affiliations:** 1 Department of Food Science and Biotechnology, Graduate School, Kyungpook National University, Daegu 41566, Korea; mbalam@knu.ac.kr; 2 Food and Bio-Industry Research Institute, Inner Beauty/Anti-Aging Center, Kyungpook National University, Daegu 41566, Korea; 3 Department of Pharmacy, Manarat International University, Dhaka 1212, Bangladesh; nscmiu@gmail.com; 4 Pharmaceutical Sciences Research Division (PSRD), BCSIR Laboratories, Dhaka 1205, Bangladesh; mhsohrab@bcsir.gov.bd; 5 Department of Pharmacy, Jahangirnagar University, Dhaka 1342, Bangladesh; sohelrana.ju@gmail.com; 6 Department of Pharmaceutical Chemistry, University of Dhaka, Dhaka 1205, Bangladesh; cmhasan@gamil.com; 7 knu BnC, Daegu 41566, Korea

**Keywords:** cerevisterol, *Fusarium solani*, *Aponogeton undulatus* Roxb., NF-κB, Nrf2, HO-1

## Abstract

As part of our continuous effort to find potential anti-inflammatory agents from endophytic fungi, a *Fusarium solani* strain, isolated from the plant *Aponogeton undulatus* Roxb., was investigated. Cerevisterol (CRVS) was identified from endophytic fungi, a *Fusarium solani* strain, and moreover exhibited anti-inflammatory activity. However, the underlying mode of action remains poorly understood. The aim of this study is to reveal the potential mechanisms of CRVS against inflammation on a molecular level in LPS-activated RAW 264.7 peritoneal macrophage cells. CRVS was isolated from *F. solani* and characterized based on spectral data analysis. The MTT assay was performed to measure cell viability in CRVS-treated macrophages. Anti-inflammatory activity was assessed by measurement of nitric oxide (NO) and prostaglandin E_2_ (PGE_2_) levels, as well as the production of various cytokines, such as tumor necrosis factor-α (TNF-α), interleukin-1β (IL-1β), and -6 (IL-6) in LPS-stimulated macrophages. RT-PCR and immunoblotting analyses were done to examine the expression of various inflammatory response genes. A reporter gene assay was conducted to measure the level of nuclear factor kappa-light-chain-enhancer of activated B cells (NF-κB) and activator protein-1 (AP-1) transactivation. CRVS suppresses the LPS-induced production of NO and PGE_2_, which is a plausible mechanism for this effect is by reducing the expression of iNOS and COX-2. CRVS also decreases the expression of pro-inflammatory cytokines, such as TNF-α, IL-6, and IL-1β. CRVS halted the nuclear translocation of NF-κB by blocking the phosphorylation of inhibitory protein κBα (IκBα) and suppressing NF-κB transactivation. The mitogen-activated protein kinases (MAPK) signaling pathways are also suppressed. CRVS treatment also inhibited the transactivation of AP-1 and the phosphorylation of c-Fos. Furthermore, CRVS could induce the nuclear translocation of nuclear factor erythroid 2-related factor 2 (Nrf2) by down-regulating Kelch-like ECH-associated protein 1 (Keap-1) and up-regulating hemeoxygenases-1 (HO-1) expression. The results suggest that CRVS acts as a natural agent for treating inflammatory diseases by targeting an MAPK, NF-κB, AP-1, and Nrf2-mediated HO-1 signaling cascade.

## 1. Introduction

Inflammation is a physiological defense response of the body to tissue injury and infection caused by wounding, microbial pathogen infections, or chemical irritation [1]. Various innate immune cells such as macrophages, fibroblasts, mast cells, and neutrophils are activated in response to infection. Among these responses, the activation of macrophages plays a pivotal role in the progression of multiple inflammatory diseases via the release of large amounts of nitric oxide (NO), prostaglandin (PG), and pro-inflammatory cytokines, such as tumor necrosis factor-α (TNF-α), interleukin-1β (IL-1β), and -6 (IL-6), and reactive oxygen species (ROS) [2,3]. Therefore, inhibiting these pro-inflammatory mediators and cytokines in activated macrophages should facilitate the treatment of inflammatory diseases.

Various signaling pathways are involved in transducing the inflammatory response. Transcription factor nuclear factor kappa-light-chain-enhancer of activated B cells (NF-κB) regulates inducible nitric oxidase synthase (iNOS), cyclooxygenase-2 (COX-2), and other pro-inflammatory cytokines in LPS-induced macrophages by binding to their promoter regions [4]. In resting cells, NF-κB is sequestered in the cytosol by its endogenous inhibitor protein κBα (IκBα). Challenges such as LPS-stimulation induce the phosphorylation of IκBα protein, triggering ubiquitin-dependent IκBα degradation in the proteasome, and resulting in rapid and transient nuclear translocation of NF-κB and the subsequent activation of specific genes [5]. Moreover, activator protein-1 (AP-1), a heterodimeric transcription factor composed of c-Fos and c-Jun, can also modulate inflammatory response genes by binding to AP-1 recognition sites [6]. Mounting evidence indicates that the mitogen activated protein kinases (MAPK) signaling cascade, consisting of c-Jun N-terminal kinases (JNK), extracellular signal-regulated kinases (ERKs), and p38 mitogen-activated protein kinases (p38) predominantly activates NF-κB and AP-1 transcription factors. Activation by phosphorylation of any of these three proteins can regulate mammalian inflammation [2,6,7]. Multiple evidences suggested that p38 has several roles in inflammation. p38 works as proinflammatory cytokine receptors downstream signaling molecule and mediates pro-inflammatory cytokine (IL-1β and IL-6) synthesis by both transcriptional and posttranscriptional regulation. p38 inhibition not only lessens production of the pro-inflammatory cytokines, but also decreases the signaling effect of these cytokines [8,9]. Furthermore, p38 deficient mice showed LPS-induced cytokine production and remained susceptible to inflammatory diseases [10]. Additionally, several evidence suggest that hemeoxygenase-1 (HO-1), which is tightly regulated by the activation of MAPK-mediated nuclear factor erythroid 2-related factor 2 (Nrf2) signaling, has a crucial role in inhibiting the production of ROS and pro-inflammatory cytokines in LPS-stimulated macrophages [11,12]. Therefore, scavenging of ROS and activating cellular anti-oxidation systems are thought to be strategies for defeating inflammation.

**Figure 1:**
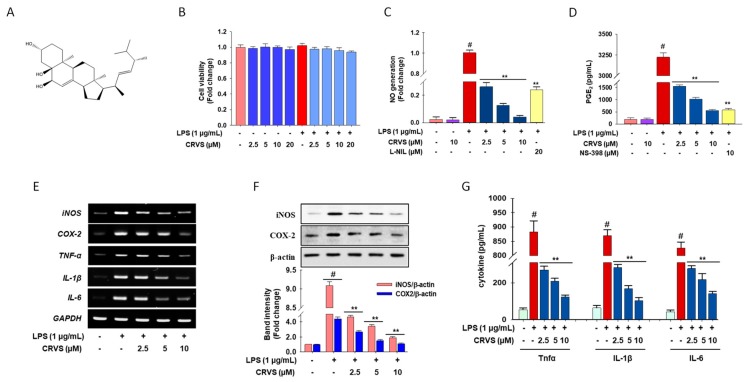
Anti-inflammatory effects of cerevisterol (CRVS). The chemical structure of CRVS (**A**). Cells (1 × 10^5^ cells/mL) were seeded in a 96-well plate and incubated for 24 h. Then the cells were treated with indicated concentrations of CRVS (2.5–20 µM) or vehicle alone for 20 h and/or for 3 h, followed by LPS (1 µg/mL) and incubated for 24 h. Effect of CRVS on cell viability (**B**), nitric oxide (NO) production (**C**), prostaglandin E_2_ (PGE_2_) (**D**), and mRNA expression of inducible nitric oxide synthase (iNOS), cyclooxygenase-2 (COX-2), tumor necrosis factor-α (TNF-α), interleukin-1β (IL-1β), and interleukin-6 (IL-6) (**E**); protein expression of iNOS and COX-2 (**F**) and the cellular expression of TNF-α, IL-1β, and IL-6 in LPS-induced RAW 264.7 macrophages (**G**). #*p* < 0.05 as compared with the vehicle-treated control; **p < 0.05 as compared with LPS alone.

Plant secondary metabolites have been a crucial source of drugs since ancient times, and recently, endophytes have become a prominent source of secondary metabolites [13]. A considerable number of phytosterols have also been isolated from fungi. Therefore, as part of our continuous effort to find potential anti-inflammatory agents from endophytic fungi, a *Fusarium solani* strain, isolated from the plant *Aponogeton undulatus* Roxb., was investigated. Chemical investigation of the ethyl acetate extract of the endophytic fungus led to the isolation of cerevisterol (CRVS) (Figure 1A, Supplementary Figures S1–S4, and Table S1) along with other known and some novel compounds [14]. Several studies propose that plant sterols and stanols may exert anti-inflammatory and anti-oxidant effects [15]. Previous studies revealed that CRVS exhibits bioactivities such as antibacterial, antioxidant, and anti-osteoporotic effects as well as the inhibition of NF-κB [16,17,18]. Therefore, we hypothesized that CRVS is a candidate anti-inflammatory agent. However, the mode of action of CRVS against inflammation has not yet been elucidated. In this study, we explore the anti-inflammatory effects and investigate the molecular mechanism of action of CRVS in LPS-stimulated macrophages.

## 2. Materials and Methods

### 2.1. Chemicals and Reagents

The isolation procedure and structural determination of cerevisterol (CRVS) from *F. solani* were described elsewhere [14]. HPLC analysis revealed a purity of ≥95%. CRVS was dissolved in dimethyl sulfoxide (DMSO) and stored at a concentration of 10 mM. This stock solution was further diluted in cell culture media to achieve a final DMSO concentration that was <0.1% *v*/*v*.

### 2.2. Cell Culture and Cell Viability Measurement

The murine macrophage RAW 264.7 cell line was obtained from the American Type Culture Collection (ATCC, Manassas, VA, USA) and cultured with Dulbecco’s modified Eagle’s medium (DMEM) (Life Technologies, Grand Island, NY, USA), supplemented with 10% fetal bovine serum (FBS) and 1% P/S (100 units/mL of penicillin and 100 μg/mL of streptomycin, Grand Island, NY, USA). The isolation of murine peritoneal macrophages was performed as described previously [19]. Briefly, macrophages were harvested at four days after intraperitoneally injection of 1 mL of 3% thioglycollate (TG) to pathogen free C57BL/6 mice (six to eight week of age). Peritoneal lavage was performed by using 8 mL of Hanks’ balanced salt solution (HBSS) containing 10 U/mL of heparin. Then, the cells were distributed in DMEM, supplemented with 10% FBS. The cells were seeded in a 96-well plate at a concentration of 1 × 10^6^ cells/mL to allow macrophage adherence by incubation for 3 h at 37 °C in a humidified incubator containing 5% CO_2_. All animals were quarantined and acclimatized to the laboratory environment for at least one week prior to the experiment under the Guidelines of the Committee on Laboratory Animal Ethics, Kyungpook National University (KNU 2018-0052, Daegu, Korea). The cell viability was measured by MTT assay as described previously [20].

### 2.3. Measurement of NO, PGE_2_, TNF-α, IL-1β, and IL-6

Cell culture supernatants were used to perform nitric oxide (NO) detection by Griess reagent [7]. Prostaglandin E_2_ (PGE_2_), tumor necrosis factor-α (TNF-α), interleukin-1β (IL-1β), and -6 (IL-6) were quantified by ELISA according to the manufacturer’s protocol.

### 2.4. Intracellular ROS Measurement

According to the method described elsewhere [20], an oxidant-sensitive fluorescent probe DCFH-DA was used to evaluate the intracellular reactive oxygen species (ROS) scavenging activity of CRVS.

### 2.5. Transfection and Luciferase Assays for NF-kB and AP-1

RAW 264.7 cells were transfected with a pNF-κB-Luc reporter gene or pAP-1-Luc plasmids (Beyotime Biotechnology, Nantong, China) for 24 h. Then, the cells were treated with CRVS for 2 h and stimulated with or without LPS treatment for another 8 h. Cell lysates were prepared for measuring luciferase activity using the Luciferase Assay System (Promega, Madison, WI, USA).

### 2.6. Transfection of Small interfering RNA (siRNA)

Cells (1 × 10^5^ cells/mL) were transfected with 10–50 nM siRNA using Lipofectamine RNAiMax (Invitrogen, Carlsbad, CA, USA) according to the manufacturer’s instructions. si-Control RNA and si-Nrf2 RNA were purchased from Santa Cruz Biotechnology (Santa Cruz, CA, USA).

### 2.7. Reverse-Transcription Polymerase Chain Reaction (RT-PCR) Analysis

TRIzol Reagent (Invitrogen Co., Carlsbad, CA, USA) was used to isolate total RNA, while cDNA was prepared by using RT & GO Mastermix (MP Biomedicals, Seoul, Korea) according to the manufacturers’ protocols. RT-PCR was carried out with various primer sequences (Supplementary Data, Table S2) using a PCR Thermal Cycler Dice TP600 (Takara Bio Inc., Otsu, Japan) [20].

### 2.8. Western Blot Analysis

Ice-cold radioimmunoprecipitation assay (RIPA) buffer was used to harvest and lyse the cells. A nuclear and cytoplasmic extraction kit (Sigma-Aldrich Co., St. Louis, MO, USA) was used to prepare nuclear and cytosolic protein extracts. Protein content was confirmed by bicinchoninic acid (BCA) protein assay kit (Pierce, Rockford, IL, USA). Protein (25 μg) adequate for Western blot analysis was isolated as described in our previous report using various antibodies (Supplementary Data, Table S3) [20].

### 2.9. Molecular Docking Study

The crystal structure of the Kelch-Neh2 complex [PDB: 4L7b] was obtained from the Protein Data Bank (PDB). (http://www.rcsb.org). The protein structures were prepared using UCSF Chimera 1.13.1 (http://www.cgl.ucsf.edu/chimera) to remove all non-receptor atoms, including water, ions, and miscellaneous compounds. The ligand (CRVS) was prepared using ChemBio3D Ultra version 12.0 (PerkinElmer, Waltham, MA, USA), and then an MMFF94 energy minimization was performed. AutoDock Vina (The Scripps Research Institute, La Jolla, CA, USA) was used for the molecular docking simulations [21]. The ligand was removed and a site sphere was specified to define the active site of KEAP1 receptor. The center of the grid box was −2.4, 2.8, and −29.21Å in x, y, and z axis, respectively, whereas the dimension was 16.21, 15.25, and 16.52Å in x, y, and z axis, respectively. The best conformation with the lowest binding energy was chosen. After the docking search was completed, it was visualized using a PyMOL Molecular Graphics System (version 1.7.4, Schrödinger, Inc., New York, NY, USA). The binding results were visualized as 3D and 2D diagrams using Discovery Studio Visualization version 4.5 (Accelrys, Inc., San Diego, CA 92121, CA, USA) and LigPlot viewer (EMBL-EBI, Wellcome Genome Campus, Hinxton, Cambridgeshire, CB10 1SD, UK, http://www.ebi.ac.uk/thornton-srv/software/LIGPLOT).

### 2.10. Statistical Analysis

GraphPad Prism Software (GraphPad Software Inc., San Diego, CA, USA) was used for the analysis of data. All data are expressed as the mean ± standard deviation (SD; n = 3) and analyzed using one-way analysis of variance (ANOVA), followed by Dennett’s test. A value of *p* < 0.05 was considered significant.

## 3. Results

### 3.1. Effects of CVRS on Cell Viability, Inflammatory Mediators, and Pro-Inflammatory Cytokines in LPS-Stimulated RAW 264.7 Macrophages

As shown in Figure 1B, CRVS treatment at 2.5, 5, 10, and 20 μM has no toxic effects. Thus, these nontoxic concentrations were used in further experiments. In comparison with the untreated control, LPS treatment significantly increases nitric oxide (NO) and prostaglandin E_2_ (PGE_2_) production by 10 fold and 12 fold, respectively (column 3 of Figure 1C,D, respectively), whereas CRVS treatment attenuates this trend significantly in a concentration-dependent manner (columns 4–6 in Figure 1C,D, respectively). Interestingly, at 10 µM, CRVS has better NO inhibitory activity than L-NIL (N6-(1-iminoethyl)-lysine, hydrochloride), a selective inducible nitric oxide synthase (iNOS) inhibitor (3.5 times). Although, CRVS has similar PGE_2_ inhibitory activity to NS-398 (*N*-[2-(cyclohexyloxy)-4-nitrophenyl]methanesulfonamide), a selective cyclooxygenase 2 (COX-2) inhibitor in PGE_2_ generation.

RT-PCR analysis indicates that mRNA levels of inducible iNOS, COX-2, tumor necrosis factor-α (TNF-α), interleukin-1β (IL-1β), and interleukin-6 (IL-6) are significantly increased in LPS-stimulated RAW 264.7 cells compared with untreated cells, whereas CRVS treatment significantly lessened this trend, as shown in Figure 1E. Likewise, immunoblotting and ELISA assays reveal that LPS-treatment significantly upregulated iNOS, and COX-2 protein by 9 fold and 4.2 fold, respectively (Figure 1F) as well as boosted the generation of cellular TNF-α, IL-1β, and IL-6 by 7 fold, 6.5 fold, and 6 fold, respectively (Figure 1G). In contrast, CRVS treatment statistical significantly inhibits the LPS-induced overexpression of iNOS and COX-2 as well as TNF-α, IL-1β, and IL-6 ((Figure 1F,G, respectively) in a concentration-dependent manner. This data suggests that CRVS inhibits the production of NO and PGE_2_ as well as pro-inflammatory cytokines by regulating their respective genes in activated RAW 264.7 macrophages.

### 3.2. Inhibition of NF-κB and AP-1 signaling by CRVS in LPS-induced RAW 264.7 Macrophages

**Figure 2:**
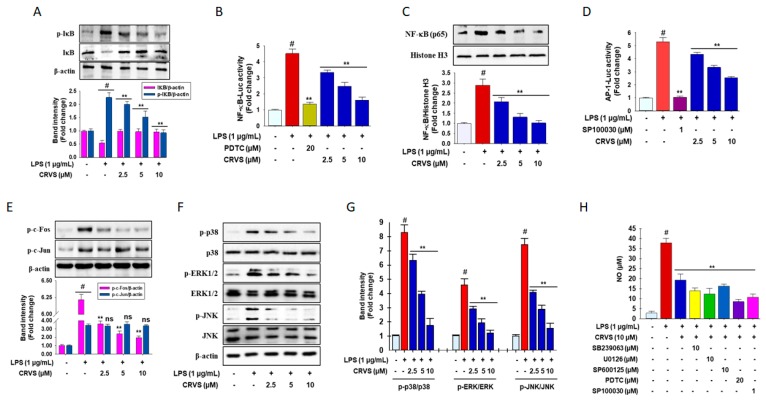
Inhibition of NF-κB and AP-1 signaling through phosphorylation of MAPK by CRVS. Phosphorylation of inhibitor protein κB (IκB) (**A**); nuclear factor kappa-light-chain-enhancer of activated B cells (NF-κB) promoter activity (**B**). Nuclear translocation of NF-κB p65 subunit (**C**) is inhibited by CRVS in LPS-stimulated RAW 264.7 cells, analyzed by Western blotting. Activator protein-1 (AP-1) promoter activity (**D**); phosphorylation of c-Fos but not c-Jun (**E**) is suppressed by CRVS in LPS-stimulated RAW 264.7 cells. Cells (1 × 10^5^ cells/mL) were seeded in a 6-well plate and incubated for 24 h. Then, the cells were treated with indicated concentrations of CRVS (2.5–20 µM) or vehicle alone followed by LPS (1 µg/mL) and incubated for 30 min. Effects of CRVS on mitogen activated protein kinases (MAPKs) (c-Jun N-terminal kinases (JNK), extracellular signal-regulated kinases (ERKs), and p38 mitogen-activated protein kinases (p38), phosphorylation in LPS-stimulated RAW 264.7 cells (**F**). Densitometric analysis of relative band intensities of proteins (**G**). NO production in the presence of various inhibitors (H). #*p* < 0.01 as compared with vehicle-treated control; **p < 0.05 as compared with LPS alone.

As shown in Figure 2A, LPS treatment markedly increases the level of phosphorylated inhibitor protein κB (IκB) by 2.2 fold, an effect that was attenuated by CRVS in a concentration-dependent manner. However, CRVS did not restore total IκBα protein levels. Interestingly, nuclear factor kappa-light-chain-enhancer of activated B cells (NF-κB) luciferase activity is enhanced in LPS-stimulated cells by 4.5 fold, whereas CRVS significantly down-regulates NF-κB transactivation in a dose-dependent fashion (Figure 2B). PDTC, a selective NF-κB inhibitor, also successfully blocks NF-κB transactivation. Furthermore, western blot analysis reveals that LPS exposure significantly increases the nuclear translocation of NF-κB (p65) by 2.8 fold compared with untreated control cells (Figure 2C, 2nd column). CRVS also attenuates the nuclear localization of NF-κB (p65) (Figure 2C, third–fifth columns).

LPS treatment significantly increases activator protein-1 (AP-1) luciferase activity by 5 fold compared with that in untreated control cells (Figure 2D, second column). CRVS markedly inhibits the transactivation of AP-1 in a concentration-dependent fashion (Figure 2D, fourth–sixth columns). SP100030, a selective AP-1 inhibitor, also inhibits AP-1 luciferase activity. In addition, Western blot analysis indicates prominent phosphorylation of c-Fos and c-Jun is achieved in LPS-stimulated RAW 264.7 cells by 6.2 fold and 3.25 fold, respectively, whereas CRVS causes a significant reduction in the level of phosphorylated c-Fos in a concentration-dependent manner but does not change the level of phosphorylated c-Jun (Figure 2E). Together, these results suggest a prospective role for NF-κB and AP-1 in the attenuation of pro-inflammatory mediators by CRVS in activated macrophages.

### 3.3. CRVS Inhibits MAPK Phosphorylation in LPS-Induced RAW 264.7 Macrophages

To further elucidate the anti-inflammatory mechanism of CRVS, the mitogen activated protein kinases (MAPKs) (c-Jun N-terminal kinases (JNK), extracellular signal-regulated kinases (ERKs), and p38 mitogen-activated protein kinases (p38) phosphorylation was analyzed by Western blotting. As indicated in Figure 2F-G, LPS stimulation triggers high levels of phosphorylated ERK, p38, and JNK by 4.5, 8.2, and 4.7 fold, respectively, whereas CRVS treatment suppresses this trend in LPS-induced RAW 264.7 cells. To investigate whether inhibition of MAPK signaling attenuates inflammatory symptoms, various inhibitors such as SB239063, U0126, and SP600125, selective inhibitors of p38, ERK and JNK, respectively, as well as PDTC and SP100030, a pharmacological inhibitor of NF-κB and AP-1, respectively, were used to determine the level of NO generation in LPS-induced RAW 264.7 cells. Interestingly, all inhibitors inhibit LPS-stimulated NO generation in LPS-induced RAW 264.7 cells. These results suggest that the anti-inflammatory effects of CRVS might be associated with down-regulation of both NF-κB and AP-1 signaling cascades through the modulation of phosphorylated MAPKs in activated macrophages.

### 3.4. CRVS Suppresses the Production of NO and Pro-Inflammatory Cytokines in LPS-Stimulated Murine Peritoneal Macrophages

**Figure 3:**
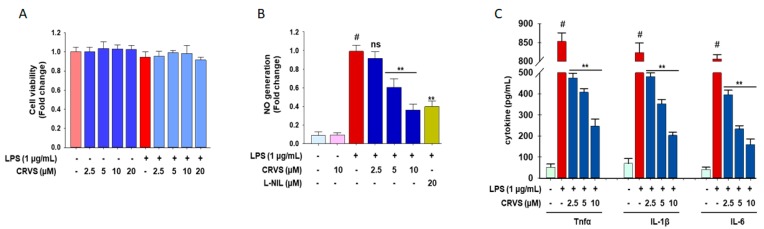
The anti-inflammatory effects of CRVS in LPS-stimulated murine peritoneal macrophages. Thioglycolate (TG)-elicited macrophages were pretreated with the indicated concentrations of CRVS (2.5, 5, 10, and 20 μM) for 1 h following treatment with LPS (1 μg/mL) for 24 h. Effects of CRVS on the cell viability (**A**); the production of NO (**B**); and TNF-α, IL-1β, and IL-6 (**C**) were examined. #*p* < 0.01 as compared with vehicle-treated control; **p < 0.05 as compared with LPS alone.

The anti-inflammatory effects of CRVS were also evaluated using LPS-stimulated murine peritoneal macrophages. The results show that CRVS does not exhibit cell toxicity up to a concentration of 20 µM (Figure 3A). As expected, CRVS alone did not affect the production of NO, whereas CRVS significantly blocks the LPS-stimulated overproduction of NO (9 fold) in LPS-stimulated murine peritoneal macrophages in a concentration-dependent manner (Figure 3B). Moreover, CRVS also suppresses the production of various pro-inflammatory cytokines such as TNF-α, IL-1β, and IL-6 in LPS-stimulated murine peritoneal macrophages in a concentration-dependent manner (Figure 3C).

### 3.5. Effects of CRVS on ROS Generation and HO-1 Expression in LPS-Induced RAW 264.7 Cells

**Figure 4:**
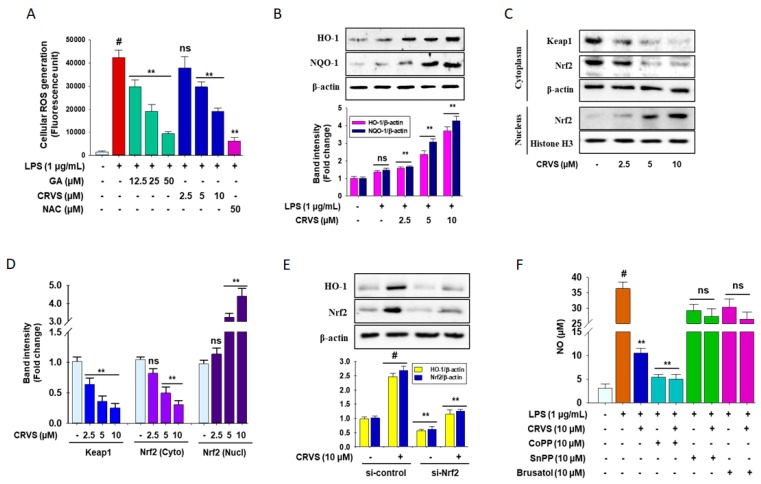
The antioxidant effects of CRVS in RAW 264.7 cells. Cells (1 × 10^5^ cells/mL) were seeded in a 6-well plate and incubated for 24 h. Then, the cells were treated with indicated concentrations of CRVS (2.5–10 µM) or vehicle alone for 24 h. Effects of CRVS on scavenging of intracellular reactive oxygen species (ROS) (**A**); the expression of heme oxygenase 1 (HO-1) and NAD(P)H quinone dehydrogenase 1 (NQO-1) (**B**); Kelch-like ECH-associated protein 1 (Keap-1), and nuclear factor erythroid 2-related factor 2 (Nrf2) in both cytosol and nuclear fractions (**C**) was detected by Western blot analysis. Densitometric analysis was carried out to assess the quantification of the relative band intensities (**D**). Cells were treated with the siRNA for Nrf2 (si-Nrf2) with and without CRVS according to the protocol described in materials and methods, and protein levels of Nrf2 and HO-1 were analyzed by immunoblotting (**E**). The production of NO was measured using a Griess reagent assay (F). #*p* < 0.01 as compared with vehicle-treated control; **p < 0.05 as compared with LPS alone.

LPS can initially increase reactive oxygen species (ROS). As shown in Figure 4A, cells stimulated with LPS have increased levels of intracellular ROS by 3.45 fold, which are reduced by CRVS treatment in a dose-dependent manner. Gallic acid and N-acetylcysteine (NAC) and potent ROS scavengers also exhibit a similar trend. The impact of CRVS on phase II detoxification enzymes was also investigated. As described in Figure 4B, Western blot analysis reveals that CRVS treatment increases the expression of heme oxygenase 1 (HO-1) and NAD(P)H quinone dehydrogenase 1 (NQO-1) in LPS-stimulated RAW 264.7 cells by 3.5 and 4.2 fold at 10 µM, respectively, in a concentration-dependent manner.

CRVS treatment significantly down-regulates Kelch-like ECH-associated protein 1 (Keap-1) expression by 4 fold at 10 µM in a dose-dependent manner (Figure 4C, upper panel, and 4D), whereas the nuclear localization of nuclear factor erythroid 2-related factor 2 (Nrf2) is markedly increased (4.5 fold at 10 µM) and associated with decreased Nrf2 levels in the cytosol (Figure 4C, middle and lower panel, and 4D). Moreover, to confirm that CRVS activates HO-1 through Nrf2, cells were transfected with small interfering RNA (siRNA) for Nrf2 (si-Nrf2) before CRVS treatment. As expected, si-Nrf2 significantly inhibits Nrf2 protein levels, and the addition of CRVS did not exhibit any further effects (Figure 4E, lower panel). HO-1 induction by CRVS was also efficiently abolished in si-Nrf2 treated cells (Figure 4E, upper panel).

Furthermore, to investigate whether inhibition of Nrf2/HO-1 signaling attenuates inflammatory symptoms, HO-1 activator and inhibitor CoPP and SnPP, respectively, and the pharmacological Nrf2 inhibitor brusatol were used to assess NO generation in LPS-induced RAW 264.7 cells. As expected, CoPP abolishes NO production, whereas SnPP and brusatol treatment halt this trend (Figure 4F). These results suggest that the anti-inflammatory effects of CRVS might be associated with augmentation of HO-1 expression through modulating a Keap-1/Nrf2 pathway in activated macrophages.

**Figure 5:**
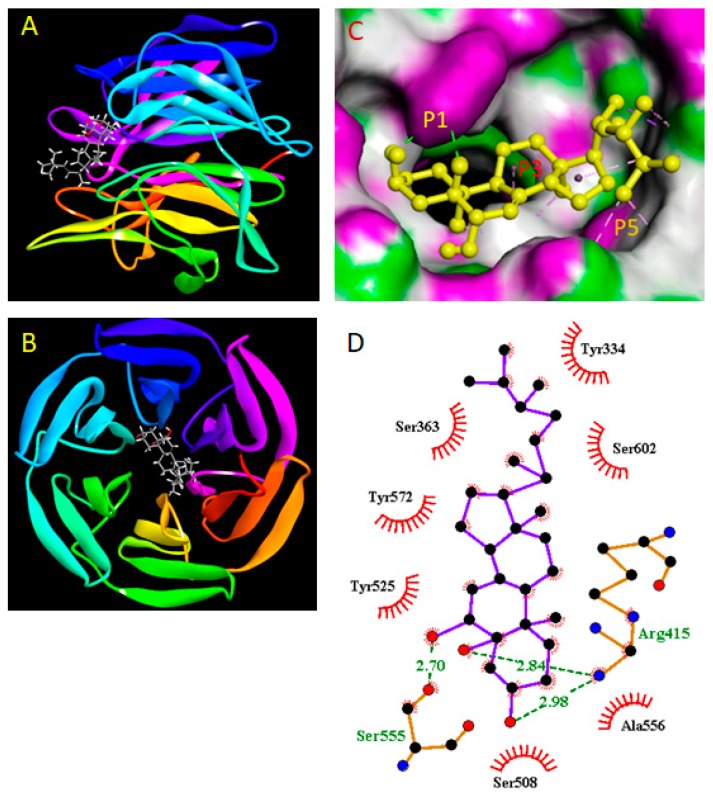
Analysis of the molecular docking between CRVS and Keap-1. Front view (**A**) and top view (**B**) of the docking mode of CRVS (azure and red) in the binding site of Keap1 (shown in ribbon representation). The binding mode of CRVS with Keap-1 in the co-crystallization form (PDB code 4L7B) (**C**). 2D binding interaction between CRVS and Keap-1 was generated by LigPlot. The green line represents the hydrogen bond interactions (**D**).

Next, to investigate whether CRVS directly inhibits the Keap-1, a molecular docking study was performed using the AutoDock Vina. The binding affinity between CRVS and Keap-1 protein (Figure 5A,B) was expressed as binding energy value that to be −9.1 kcal/mol, whereas the original ligand (IVV; (1*S*,2*R*)-2-{[(1*S*)-1-[(1,3-dioxo-1,3-dihydro-2H-isoindol-2-yl)methyl]-3,4-dihydroisoquinolin-2(1H)-yl]carbonyl}cyclohexanecarboxylic acid) bind with Keap-1 was shown −10.0 kcal/mol. It was found to interact specifically with the P1, P3, and P5 subpocket of Keap-1 receptor (Figure 5C). The hydroxy groups at positions 3, 5, and 6 of the steroid structure of CRVS were observed to interact via hydrogen bond with Arg415 and Ser555 with a bond distance (Å) of 2.84, 2.98, and 2.70, respectively. Furthermore, the molecule was stacked in a conformation that allows it to interact with P3 and P5 subpocket amino acid residues such as Ala556, and Ser602 (for P3 subpocket) and Try334, Try525, and Try572 (for P5 subpocket) residues via the hydrophobic interactions [22]. Thus, these simultaneous binding of CRVS with the P1, P3, and P5 subpocket of Keap-1 and the strong binding energy suggested the protein to be its direct binding target. This interaction pattern is also similar to that of the original ligand (Supplementary data Figure S5), demonstrating that CRVS has a high potential inhibitory activity.

## 4. Discussion

Recent evidence suggests that plant sterols and stanols exert beneficial effects against inflammation and oxidative stress [15]. The present study demonstrates that cerevisterol (CRVS) regulates the inflammatory response in mouse peritoneal macrophages.

Activated macrophages play a pivotal role in the regulation of inflammatory processes [23] and causes mass production of inflammatory mediators such as NO and PGE_2_, as well as pro-inflammatory cytokines such as TNF-α, IL-1β, and IL-6, leading to the progression of various inflammatory disorders [24]. In the present study, LPS-induced NO and PGE_2_ were strongly suppressed by CRVS without having cytotoxic effects. NO and PGE_2_ are primarily derived from L-arginine and arachidonic acid by the action of iNOS and COX-2, respectively. The inhibition of iNOS and COX-2 can exert significant immunosuppressive effects [2,25]. Our results show that CRVS blocks the LPS-stimulated up-regulation of iNOS and COX-2, signifying that the degree of inhibition of NO and PGE_2_ by CRVS is associated with the down-regulation of iNOS and COX-2 expression, respectively. Moreover, a series of pro-inflammatory cytokines are generated in macrophages by LPS stimulation and are involved in initiating and regulating the inflammatory process [4]. Accordingly, the suppression of these pro-inflammatory cytokines could be a potential target for anti-inflammatory drug development. Our study revealed that CRVS attenuated both the expression of mRNA and the protein levels of TNF-α, IL-1β, and IL-6 in LPS-induced macrophages. Our studies are also in line with previous studies [26,27], in which CRVS treatment significantly suppressed the production of NO and PGE_2_ by modulating their respective gene iNOS and COX-2 at their transcriptional and translational level, respectively, in LPS-induced RAW 264.7 cells. In addition, our studies also revealed that CRVS have the potential to lessen the inflammatory symptoms in LPS-induced primary murine peritoneal macrophages (Figure 3) which is the first time reported ever. Although CRVS can lessen the inflammatory symptoms by reducing the generation of inflammatory mediators as well as block the production of pro-inflammatory cytokines [26,27], but the underling mechanism is still poorly understood. Thus, we hypothesized that CRVS may have the potential to regulate the inflammatory transcription factors and showed its anti-inflammatory effects.

Mounting evidence suggests that transcription factors such as NF-κB and AP-1 are tightly involved in regulating inflammation through the activation of diverse inflammatory genes, including iNOS, COX-2, TNF-α, IL-1β, and IL-6 [3]. NF-κB activation in inflammatory cells depends on the phosphorylation-induced protein degradation of the inhibitor of NF-κB protein (IκBs), which sequesters inactive NF-κB dimers in the cytosol of resting cells [5]. In this study, LPS activates the NF-κB pathway by inducing nuclear translocation of NF-κB by decreasing the phosphorylation of IκBα, whereas CRVS blocked this trend. CRVS treatment also attenuated the LPS-stimulated AP-1 activation. Furthermore, CRVS significantly inhibited LPS-mediated NF-κB and AP-1 transactivation. Our findings demonstrated that the suppression of pro-inflammatory mediators and cytokines by CRVS was at least in part associated with inhibition of NF-κB p65 and AP-1 activation and transactivation. NF-κB and AP-1 activation are governed by MAPKs that play key roles in inflammatory responses [4,19]. Recent research has also shown that several small molecules can regulate the expression of iNOS, COX-2, and cytokine genes through the MAPK signaling pathway [28]. Our studies also revealed that CRVS attenuate the phosphorylation of p38, JNK, and ERK. Furthermore, the MAPK inhibitors SB239063, SP600125, and U0126 suppressed the production of NO. These findings elucidated the fundamental role of MAPKs in the anti-inflammation effect of CRVS.

ROS are participants in LPS-induced inflammation by activating many signaling pathways [29]. Moreover, recent studies have reported that up-regulation of HO-1 expression causes anti-inflammatory effects and protects oxidative stress-induced cell death [30]. This study indicates that CRVS attenuated ROS production in LPS-treated cells while enhancing the expression of HO-1. Interestingly, pretreatment with HO-1 inducer CoPP distinctly repressed LPS-stimulated NO generation, whereas pretreatment with SnPP, an HO-1 inhibitor, drastically reversed this trend with or without CRVS. HO-1 expression is closely regulated by Nrf2. Nrf2 plays defensive and repair roles in cells. Activation of Nrf2 by external stimuli can modulate antioxidant and anti-inflammatory factors [31]. Previous studies have confirmed that resveratrol inhibits the activities of NF-κB and AP-1 and boosts detoxification and antioxidant enzymes through an Nrf2 signaling pathway [32]. Nevertheless, in resting cells, Nrf2 is sequestered in the cytoplasm by Keap-1. Following oxidative stress, Keap-1 releases Nrf2, resulting in nuclear translocation of Nrf2 [20]. Consistent with these findings, CRVS down-regulated the expression of Keap-1 and induced the nuclear translocation of Nrf2. Sulforaphane, derived from natural sources, is a strong Nrf2 activator that functions by modifying Keap-1 cysteine residues [28]. Molecular docking analysis was performed with CRVS to assess its binding with Keap-1 protein. A previous study reported that Nrf2 was inserted into the hydrophobic region of the Keap-1 pocket [33]. The binding cavity of Keap-1 is divided into five subpockets as P1, P2, P3, P4, and P5. Among them, P1 and P2 play major role in powerful binding interactions and provide to total binding free energy. The amino acid residues of P1 subpocket are Ser508, Phe478, Ile461, Arg483, Arg415, and Gly462, which is highly positively charged and the electrostatic interactions with the Arg483 and Arg415 are significant for binding. The peptide backbone occupies the P3 subpocket which is composed of Gly509 Ala556, Ser555, Ser602, Gly603, and Gly571 [22]. Molecular docking studies predicted significant hydrogen bond, and hydrophobic interactions between CRVS and residues in the subpocket P1, P3, and P5 of the Keap-1 receptor, with binding energy value of −9.1 kcal/mol. It was worth noticing that Keap-1 residues Arg415, Ser555, Ala556, and Ser602 were found to be involved in interaction with CRVS in our study, which have been reported in structural studies to be involved in Nrf2- Keap-1 binding by forming hydrogen bonds with Glu78 of Nrf2 [34]. Moreover, these docking results showed that CRVS formed multiple hydrogen and hydrophobic interactions with Keap-1 and may explain the competition and inhibition of CRVS against Keap-1-binding Nrf2. These findings demonstrated the contribution of Nrf2-induced HO-1 expression to the anti-inflammatory effects of CRVS.

## 5. Conclusions

**Figure 6:**
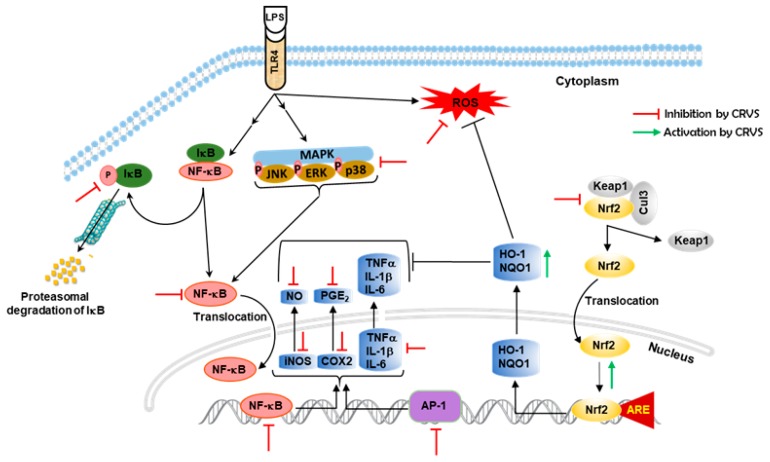
Schematic diagram of the mode of action of CRVS against inflammation in LPS-induced macrophages.

The present study furnished initial evidence that cerevisterol (CRVS), isolated from the endophytic fungal strain *Fusarium solani* exerted anti-inflammatory effects in LPS-induced macrophages via the inhibition of inflammatory mediators and their associated genes, as well as a reduction in pro-inflammatory cytokines, deactivated NF-κB and AP-1 transcription factors through the down-regulation of MAPK signaling, and, at least in part, the up-regulation of Nrf2-mediated HO-1 expression (Figure 6). This study strongly supports the potential of CRVS as a novel anti-inflammatory agent, whereas further systemic analyses are needed in LPS-challenged animals to determine the in vivo efficacy and pharmacokinetics of CRVS.

## Supplementary Materials

The supplementary materials are available online at https://www.mdpi.com/2218-273X/10/2/199/s1.

## Abbreviations

2D: two-dimensional; 3D: three-dimensional; AP-1: Activator protein-1; CRVS: cerevisterol; COX-2: cyclooxygenase; ERK: extracellular signal-regulated kinase; HO-1: heme oxygenase-1; IκBα: inhibitory κBα; iNOS: inducible nitric oxide synthase; IL-1β: interleukin 1β; IL-6: interleukin 6; IL-10: interleukin 10; JNK: c-Jun N-terminal kinase; Keap-1: Kelch ECH-associated protein-1; LPS: lipopolysaccharide; MAPK: mitogen-activated protein kinase; MTT: 3-(4,5-dimethylthiazol-2-yl)-2,5-diphenyl-tetrazoliumbromide; NF-κB: nuclear factor-κB; NO: nitric oxide; NQO-1: NAD(P)H quinone dehydrogenase-1; Nrf2: nuclear factor erythroid 2-related factor 2; PCR: polymerase chain reaction; PG: prostaglandins; PGE_2_: prostaglandin E_2_; P/S: solution of penicillin and streptomycin used for cell culture; ROS: reactive oxygen species; RT-PCR: reverse transcription PCR; TNF-α: tumor necrosis factor-α.
