# Supplementary material for: Cerevisterol Alleviates Inflammation via Suppression of MAPK/NF-κB/AP-1 and Activation of the Nrf2/HO-1 Signaling Cascade
Source: Biomolecules. 2020 Jan 29;10(2):199. doi: 10.3390/biom10020199 (PMC7072429; doi:10.3390/biom10020199)
Supplement: Supplementary file 1 [file biomolecules-10-00199-s001.pdf]

Cerevisterol Alleviates Inflammation via Suppression of MAPK/NF-κB/AP-1 and Activation of the Nrf2/HO-1 Signaling Cascade

Md Badrul Alam [1,2,†], Nargis Sultana Chowdhury [3,†], Md Hossain Sohrab [4], Md Sohel Rana [5], Choudhury Mahmood Hasan [6] and Sang-Han Lee [1,2,7,*]

[1] Department of Food Science and Biotechnology, Graduate School, Kyungpook National University, Daegu 41566, Korea
[2] Food and Bio-Industry Research Institute, Inner Beauty/Anti-Aging Center, Kyungpook National University, Daegu 41566, Korea
[3] Department of Pharmacy, Manarat International University, Dhaka 1212, Bangladesh
[4] Pharmaceutical Sciences Research Division (PSRD), BCSIR Laboratories, Dhaka 1205, Bangladesh
[5] Department of Pharmacy, Jahangirnagar University, Dhaka 1342, Bangladesh
[6] Department of Pharmaceutical Chemistry, University of Dhaka, Dhaka 1205, Bangladesh
[7] knu BnC, Daegu 41566, Korea
* Correspondence: sang@knu.ac.kr; Tel.: +82-53-950-7754; Mobile: +82-10-2537-7659; Fax: +82-53-950-6772
† These authors contributed equally to the study.

Materials and Methods

Chemicals and reagents

LPS (Escherichia coli 055:B5), N6-(1-iminoethyl)-lysine, hydrochloride (L-NIL), N-[2-(cyclohexyloxy)-4-nitrophenyl] methanesulfonamide (NS-398), Hanks' balanced salt solution (HBSS) and 3-(4,5-dimethylthiazol-2-yl)-2,5-diphenyltetrazolium bromide (MTT) were purchased from Sigma (St. Louis, MO, USA). Dulbecco's modified Eagle's medium (DMEM), fetal bovine serum (FBS), and penicillin and streptomycin were obtained from Invitrogen-Gibco (Grand Island, NY, USA). Enzyme-linked immunosorbent assay (ELISA) kits of mouse TNF-α, IL-1β, and IL-6 were provided by Biolegend (San Diego, CA, USA). The HO-1 inducer CoPP and inhibitor SnPP, and the Nrf2 inhibitor brusatol were obtained from Santa Cruz Biotechnology (San Diego, CA, USA). The NF-κB inhibitor ammonium pyrrolidine dithiocarbamate (PDTC), and AP-1 inhibitor SP100030 were purchased from R&D Systems, Inc. (Minneapolis, MN, USA).

Isolation and Characterization of cerevisterol

In this study, we investigated the fungal endophyte *Fusarium solani* isolated from the roots of *Aponogeton undulates* Roxb. growing in the deep water of Bangladesh. Cerevisterol (CRVS) (Figure S1) was obtained as colourless, needle-like crystal (25 mg) from a sub-column fraction with the polar eluent system 5% MeOH in EtOAc (v/v). It appeared as a dark quenching spot on the TLC plate (R_f = 0.23, $CHCl_3$ / 20 % MeOH) under UV light at 254 nm and also exhibited blue fluorescence at 365 nm. Compound CRVS is soluble in $CHCl_3$ and MeOH.

The ^1H NMR (400 MHz, $CDCl_3$) and ^{13}C NMR (100 MHz, $CDCl_3$) data of CRVS (Table S1, Figure S2-S3) proved the presence of 28 carbon-atom signals corresponding to two tertiary methyl, four secondary methyl, seven methylene, eleven methine and four quaternary carbons (Table S1).

In the mass spectrum of CRVS (Figure S4), the fragment ion peaks were very similar to those of an ergosterol derivative. An accurate mass measurement of CRVS obtained by HR-ESIMS yielded a parent mass at *m/z* 453.3349 in a positive ionization mode that, in conjunction with the data of the NMR spectrum, suggested the sodium adduct $[M + Na]^+$ of a compound with molecular formula $C_{28}H_{46}O_3$ (calculated for $C_{28}H_{46}O_3Na$, 453.3349).

^1H NMR and ^{13}C NMR data of compound CRVS indicates that it is structurally related to cerevesterol isolated from the fruiting bodies of Ascomycetes *Tuber indicum* [1]. These facts indicated that compound CRVS is (22*E*, 24*R*)-ergosta-7, 22-dien-3*β*, 5*α*, 6*β*-triol, confirmed by direct comparison with literature data. Cerevisterol is reported for the first time from the species *Fusarium solani*.

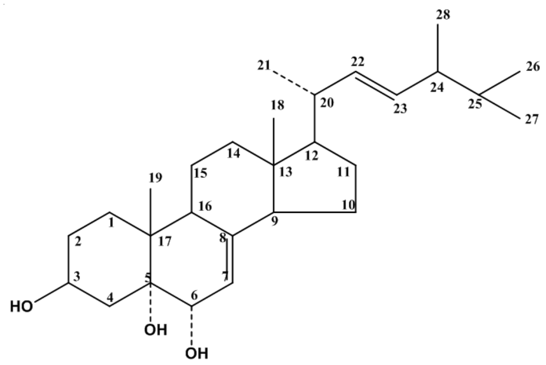

Figure S1: Structure of cerevisterol.

Table S1: ^{1}H (400 MHz, $CDCl_3$) and ^{13}C (100 MHz, $CDCl_3$) NMR data for cerevisterol

position	δ_H, (mult., *J* in Hz)	δ_C, type
1	5.14, dd (15, 7)	32.9, CH_2
2		33.1, CH_2
3	4.07, m	67.7, CH
Hax-4	2.13, q (12.2)	42.9, CH_2
Heq-4	1.76, dd (16.4 & 3.6)	
5		73.7, C
6	3.61, br.s	75.9, CH
7	5.34, br.s	117.5, CH
8		144.0, C
9		43.5, CH
10		39.2, C
11		22.1, CH_2
12		39.4, CH_2
13		43.8, C
14		54.9, CH
15		22.9, CH_2
16		30.9 CH_2
17		56.0, CH
18	0.58, s	12.4, CH_3
19	1.07, s	18.6, CH_3
20		40.4, CH
21	1.01, d (6.4)	20.0, CH_3
22	5.14, dd (15.2 & 7.4)	135.3, CH
23	5.22, dd (15.2 & 7.4)	132.2, CH
24		42.8, CH
25		33.1, CH
26	0.81, d (6.4)	21.1, CH_3

27	0.82, d (6.4)	19.8, CH_3
28	0.90, d (6.8)	17.6, CH_3

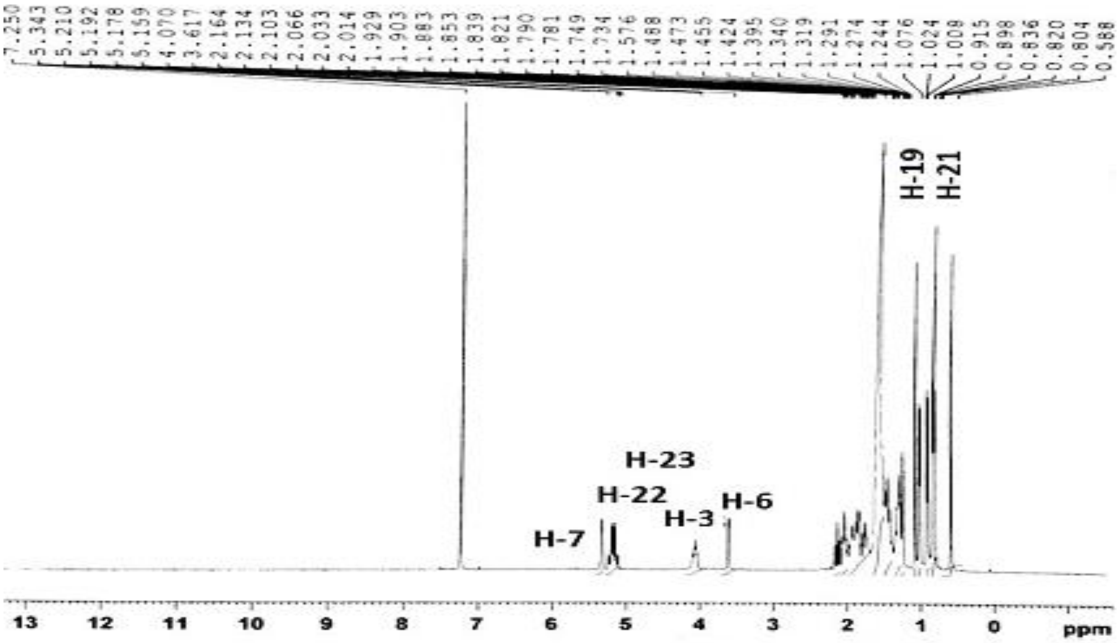

Figure S2: ^{1}H NMR spectrum (400 MHz, $CDCl_3$) of cerevisterol.

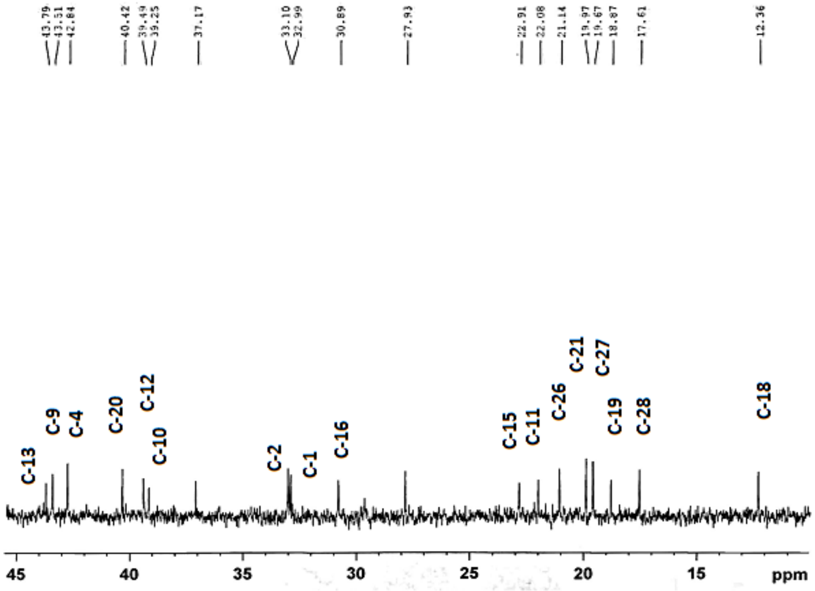

Figure S3: ^{13}C NMR spectrum (100 MHz, $CDCl_3$) of cerevisterol.

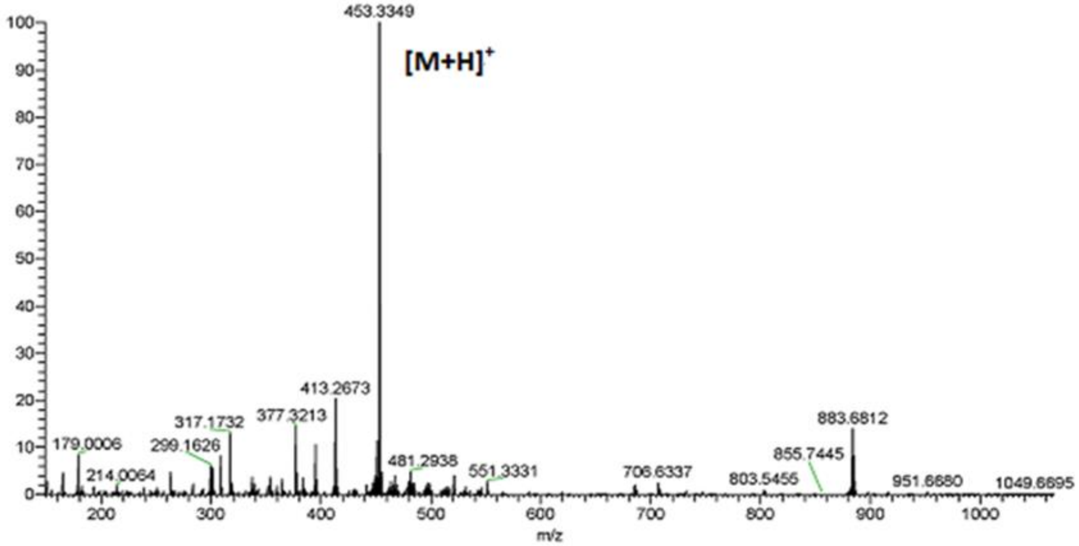

Figure S4: HRMS spectrum of cerevisterol.

Table S2: List of the primer sets used in the study

Gene name		***Sequences***
iNOS	*forward*	*GGCTGTCAGAGCCTCGTGGC*
	reverse	*CCCTTCCGAAGTTTCTGGCA*
COX2	*forward*	*AACACAGCTACGAAAACC*
	reverse	*CACAGTATGATGTAACAGT*
TNF-α	*forward*	*GGCAGGTCTACTTTGGAGTCA*
	reverse	*ACATTCGAGGCTCCAGTGAAT*
IL-1β	*forward*	*ATGGCAACTGTTCCTGAACTC*
	reverse	*CAGGACAGGTATAGATTCTTT*
IL-6	*forward*	*GAGGATACCACTCCCAACAGA*
	reverse	*AAGTGCATCATCGTTGTTCATA*
Gapdh	*forward*	*TTGTGATGGGTGTGAACCAC*
	reverse	*ACACATTGGGGGTAGGAACA*

Table S3: List of the primary antibodies used in the study

**Name**	**Catalog no.**	**Company**	**Antigen**	**Host**
Anti-iNOS	MAB9502	R&D systems	iNOS	Mouse
Anti-COX2	AF4198	R&D systems	COX2	Goat
Anti-HO-1	sc-136256	Santa Cruz Biotechnology, Inc.	HO-1	Mouse
Anti Nrf2	sc-81342	Santa Cruz Biotechnology, Inc.	Nrf2	Mouse
Anti-p-IκB-α	sc-8404	Santa Cruz Biotechnology, Inc.	IκB-α	Mouse
Anti-IκB-α	sc-373893	Santa Cruz Biotechnology, Inc.	IκB-α	Mouse
Anti-NF-κB (p65)	BS1254	Bioworld Technology, Inc.	NF-κB (p65)	Rabbit
Anti-p-c-Jun	BS4050	Bioworld Technology, Inc.	c-Jun (Y-170)	Rabbit
Anti-p-c-Fos	sc-81485	Santa Cruz Biotechnology, Inc.	c-Fos (S374)	Mouse
Anti-Histone H3	BS1405	Bioworld Technology, Inc.	Histone H3	Rabbit
Anti-p-p38	sc-166182	Santa Cruz Biotechnology, Inc.	p38	Mouse
Anti-p38	BS3567	Bioworld Technology, Inc.	p38	Rabbit
Anti-p-ERK1/2	sc-7383	Santa Cruz Biotechnology, Inc.	ERK	Mouse
Anti-ERK1/2	BS 6472	Bioworld Technology, Inc.	ERK	Rabbit
Anti-p-JNK	BS 4322	Bioworld Technology, Inc.	JNK	Rabbit
Anti-JNK	sc-7345	Santa Cruz Biotechnology, Inc.	JNK	Mouse
Anti- β-actin	Sc-47778	Santa Cruz Biotechnology, Inc.	β-actin	Mouse
Anti-NQO1	sc-271116	Santa Cruz Biotechnology, Inc.	NQO1	Mouse
Anti-Keap-1	sc-365626	Santa Cruz Biotechnology, Inc.	Keap1	Mouse

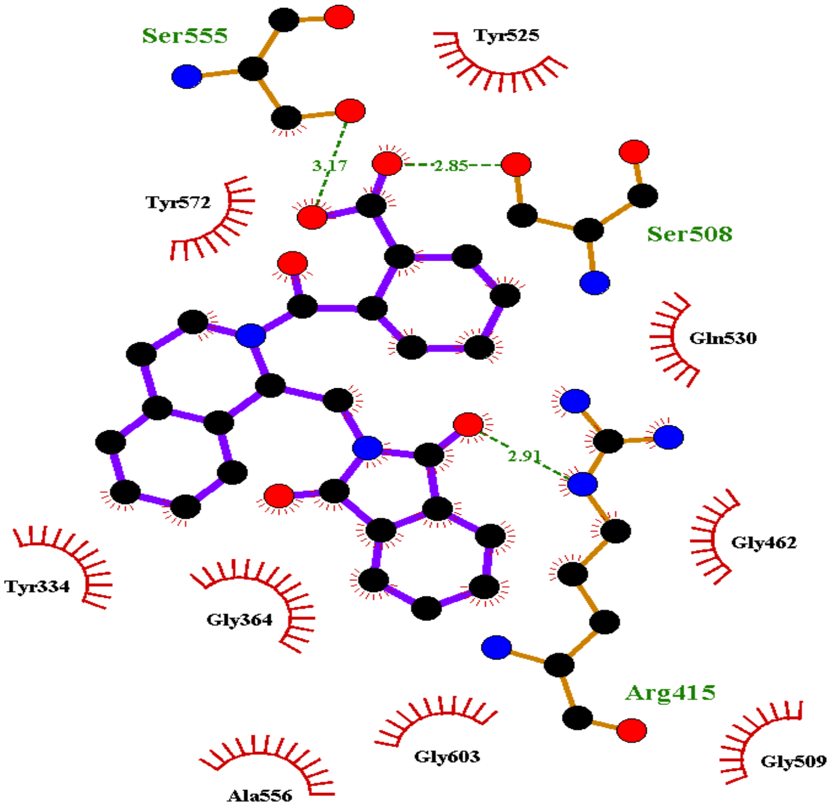

Figure S5: The molecular docking analysis of IVV with Keap-1. Binding mode of IVV (default ligand binding with Keap-1) and Keap-1 in the co-crystallization form (PDB code 3L7B) (C). 2D binding interaction between IVV and Keap-1 was generated by LigPlot. The green dotted lines represent the hydrogen bond interaction (D).

References

1. Jinming, G.; Lin, H.; Jikai, L. A novel sterol from Chinese truffles *Tuber indicum*. *Steroids* **2001**, *66*, 771–775.
